# Association between preoperative white blood cell counts and thirty-day surgical mortality after craniotomy in adult intracranial tumor patients

**DOI:** 10.3389/fneur.2024.1394568

**Published:** 2024-07-05

**Authors:** Zhichao Gao, Cheng Huang, Shengjie Fang, Jiaqing Guan, Weifeng Dong

**Affiliations:** ^1^Department of Neurosurgery, The First People's Hospital of Xiaoshan District, Hangzhou, Zhejiang, China; ^2^Department of Coloproctology, The First People's Hospital of Xiaoshan District, Hangzhou, Zhejiang, China

**Keywords:** white blood cell, thirty-day mortality, non-linear, intracranial tumor, craniotomy, cohort study

## Abstract

**Objective:**

White blood cell (WBC) counts has been identified as a prognostic biomarker which frequently predict adverse outcomes and mortality risk in various conditions. However, evidence for the association between WBC counts and short-term outcomes after intracranial tumor resection remains limited. This study aimed to explore associations between preoperative WBC counts and thirty-day surgical mortality after craniotomy in adult intracranial tumor patients.

**Methods:**

This retrospective cohort study performed secondary analysis of 18,049 intracranial tumor craniotomy patients from the ACS NSQIP database (2012–2015). The major exposure and outcome were preoperative WBC counts and thirty-day surgical mortality, respectively. Cox regression modeling assessed the linear association between them. Non-linear associations between them were evaluated by conducting smooth curve fitting using an additive Cox proportional hazard model in conjunction with segmented linear regression modeling. Subgroup analysis and interaction testing assessed effect modification. Sensitivity analysis evaluated result robustness.

**Results:**

The total thirty-day surgical mortality after craniotomy was 2.49% (450/18,049). The mean of preoperative WBC counts was 9.501 ± 4.402 × 10^9/L. Fully adjusted model shows that elevated preoperative WBC counts was independently associated with increased thirty-day surgical mortality (HR = 1.057, 95%CI: 1.040, 1.076). Further analysis revealed a non-linear association between them: below a WBC threshold of 13.6 × 10^9/L, higher WBC counts elevated thirty-day mortality (HR = 1.117; 95%CI: 1.077, 1.158), while risk plateaued and no significant mortality rise occurred above this level (HR = 1.015, 95%CI: 0.982, 1.050). Steroid usage status has a significant effect modification on the WBC-mortality association (*P* for interaction = 0.002). The non-linear WBC-mortality association was only present for non-steroid users (HR = 1.158, 95%CI: 1.108, 1.210) but not steroid users (HR = 1.009, 95%CI: 0.966, 1.055). The sensitivity analysis confirmed the result robustness.

**Conclusion:**

Elevated preoperative WBC counts were independently and non-linearly associated with an increased risk of thirty-day surgical mortality in adult non-steroid use patients undergoing craniotomy for intracranial tumors. As a convenient predictor, preoperative WBC data allows improved risk profiling and personalized management in adult intracranial tumor patients.

## Introduction

Intracranial tumors refer to abnormal cell growths in the brain or central spinal cord. Intracranial tumors significantly impact global health, responsible for 2% of all cancer deaths and the foremost reason for cancer fatalities among children (1, 2). Intracranial tumor occurs at a rate of 21 per 100,000 population, representing approximately 2% of all human cancer, with rates increasing in recent years (3, 4). Surgical resection by craniotomy is one of primary treatments for intracranial tumors. However, the potential postoperative complications and mortality risks should be considered (5, 6). Studies typically evaluate outcomes and complications during the short-term postoperative period of 30 days, with thirty-day mortality serving as an indicator of surgical risk and safety (7, 8). A study of 8,663 craniotomy patients in China found a 2.3% thirty-day surgical mortality rate for brain tumors (9), consistent with a Norwegian study (7). Meanwhile, a study from American College of Surgeons National Surgical Quality Improvement Program (ACS NSQIP) reported a 2.6% thirty-day mortality rate after craniotomy in 18,642 patients with primary brain malignancies (10). Current studies have identified several perioperative factors linked to thirty-day mortality after intracranial tumor resection, namely preoperative platelet count, hematocrit, serum sodium, blood urea nitrogen, body mass index, as well as sepsis and septic shock after surgery (10–15).

White blood cells (WBC) or leukocytes, originating from hematopoietic stem cells, are critical elements of the innate and adaptive immune system. These cells are categorized into various subsets, including neutrophils, lymphocytes, monocytes, eosinophils, and basophils, each with distinct functions in immune surveillance and response (16). As the most abundant leukocyte, neutrophils comprise 50–70% of all WBCs and are essential for acute inflammatory responses and pathogen clearance (17). An elevated WBC count, or leukocytosis, can be indicative of an ongoing inflammatory process, infectious disease, or hematological malignancy (18). Many studies have substantiated WBC counts’ prognostic value, highlighting their association with poorer outcomes in cardiovascular disease, cerebrovascular accidents, malignancies, and trauma (19–22). Meanwhile, elevated WBC counts also has been established as a prognostic indicator of surgical mortality and morbidity, associated with increasing mortality across different surgical contexts (23–25). The pathophysiological mechanism may involved is thought to be the WBC-driven inflammatory pathways that contribute to tissue injury and disease progression (26). Additionally, WBC levels also associate with genetic factors and lifestyle habits such as smoking (27). In summary, the WBC count serves as an important biomarker to assess immune function and inflammatory status *in vivo*, as well as a prognostic marker to predict mortality risk across diverse surgical and medical contexts.

To date, little research has examined associations between preoperative WBC counts and thirty-day surgical mortality in intracranial tumor patients. A study from the ACS NSQIP database showed that patients with sepsis and septic shock postsurgery has significantly higher thirty-day surgical mortality than others, with systemic inflammatory response syndrome (SIRS) as a major risk factor (10). However, the link between preoperative WBC counts, a key biomarker of inflammatory status, and thirty-day surgical mortality in intracranial tumor patients remains to be investigated. Therefore, we aimed to perform a secondary analysis based on the ACS NSQIP database to explore associations between preoperative WBC counts and thirty-day surgical mortality after craniotomy in adult intracranial tumor patients. This study may provide insights into the utility of preoperative WBC counts for prognostication after intracranial tumor resection.

## Materials and methods

### Study design and data source

This study is a retrospectively designed cohort analysis grounded on the ACS NSQIP database (2012–2015) that originally uploaded and made public by Zhang et al. (10). We performed a secondary analysis of these data (Data source: Sepsis and septic shock after craniotomy: Predicting a significant patient safety and quality outcome measure https://doi.org/10.1371/journal.pone.0235273). The original research was openly published under a Creative Commons Attribution License permitting unrestricted utilization given appropriate attribution. Thus, secondary analysis of these data does not infringe on author copyright.

### Study population

**Figure 1:**
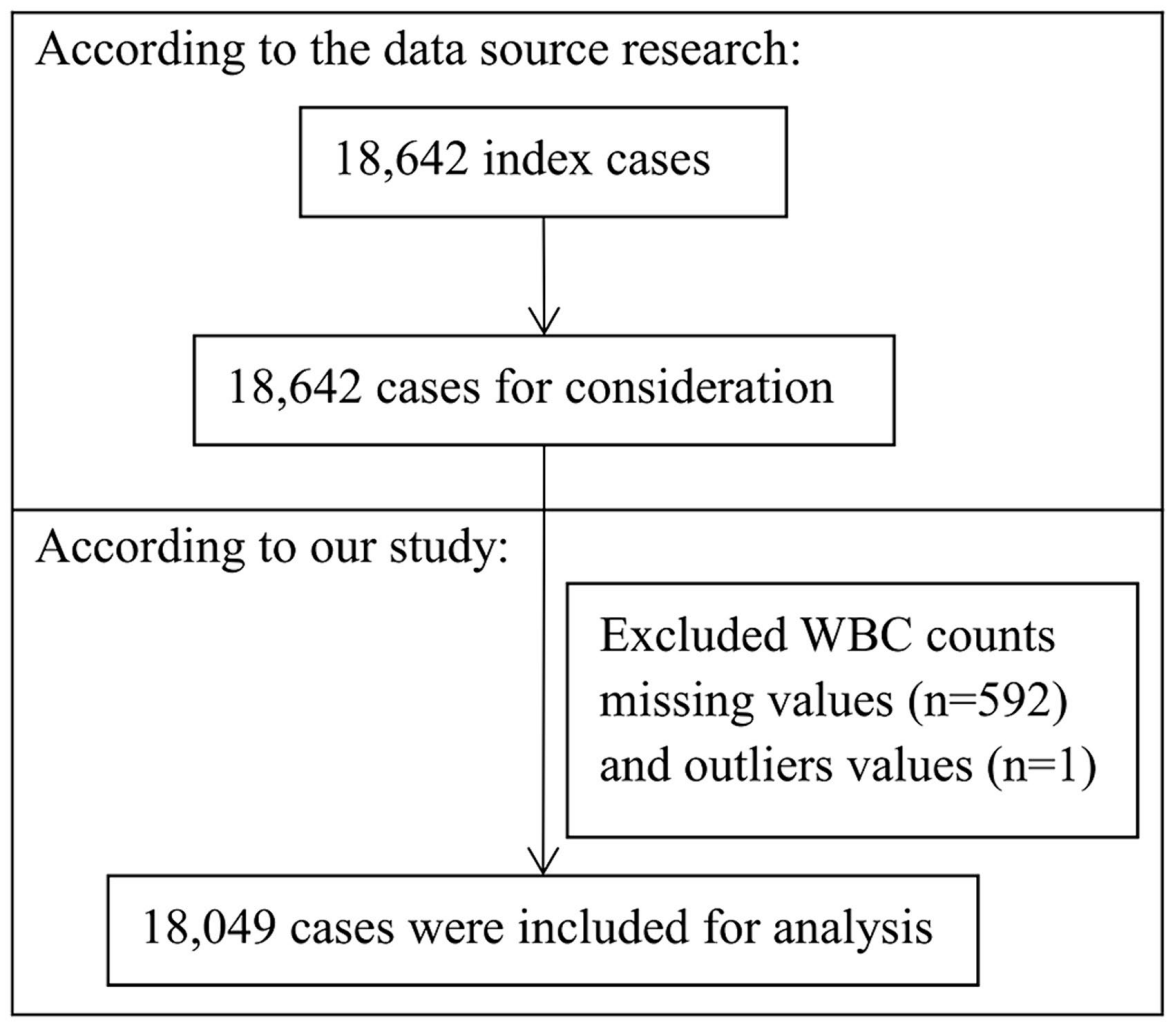
Flowchart of study population selection.

The original study analyzed 18,642 adult intracranial tumor patients undergoing craniotomy across about 400 academic and community hospitals in the United States (2012–2015). All patients were subjected to a thirty-day postoperative follow-up period. After excluding 592 patients missing preoperative WBC data and 1 patient with outliers (WBC = 119.8 × 10^9/L), 18,049 patients were retained for the analytical process (as shown in Figure 1). Informed consent was waived given the retrospective analysis and anonymous data.

### Variables

Preoperative WBC count (×10^9/L), the independent variable, was recorded as a continuous variable in the original study. The dependent variable was thirty-day surgical mortality, defined as death occurred within 30 days following craniotomy, which was tracked for 30 days in the ACS NSQIP database (10).

Selection of covariates was informed by existing literature and clinical expertise. Analyzed covariates included: (1) Continuous variables: body mass index [BMI, computed as weight/height squared (kg/m^2^)], preoperative laboratory parameters [serum sodium (Na), blood urea nitrogen (BUN), creatine (Cr), platelet (PLT) count, hematocrit (HCT), international normalized ratio (INR)], and operation time; (2) categorical variables: sex, race, age ranges, smoking status, surgical site, functional health status, severe chronic obstructive pulmonary disease (COPD), diabetes, hypertension, congestive heart failure (CHF), renal failure, dialysis, disseminated cancer, steroid use for chronic condition, pre-operation systemic infection, emergency case. See original study for more details (10).

### Statistical analysis

The percentage of missing data was: BMI 3.97% (*n* = 716); Na 2.24% (*n* = 404); BUN 6.22% (*n* = 1,123); Cr 1.99% (*n* = 359); HCT 0.07% (*n* = 12); PLT 0.11% (*n* = 19); INR 12.88% (*n* = 2,324); operation time 0.01% (*n* = 2). The missing values were substituted with their median (non-normal distribution) or mean (normal distribution) values to facilitate analysis.

Participants were stratified into quartiles by WBC counts. Continuous variables with normal distributions were expressed in terms of mean and standard deviation (SD); those non-normally distributed were reported using median values and interquartile ranges. Categorical variables were presented as counts and corresponding percentages. Differences across WBC quartiles were compared by One-way ANOVA (means), Kruskal–Wallis *H* test (medians), and chi-square test (percentages).

Univariate regression analysis by Cox proportional hazard modeling examined the relationship between baseline characteristics of study subjects and outcomes. Multivariate regression analysis by Cox proportional hazard modeling evaluated the independent impact of preoperative WBC counts on thirty-day surgical mortality risk. A confounding factor is defined as a covariate that, when introduced into the basic model or excluded from the full model, alters the regression coefficient for the exposure variable by >10% or has a regression coefficient with the outcome associated with a *p* < 0.1 (28).

Following guidelines of the Strengthening the Reporting of Observational Studies in Epidemiology (STROBE) statement (29), three distinct models were established: Crude model without any adjustments; Model I with minimal adjustments for sex and age; Model II with full adjustments for sex, age, functional health status, COPD, diabetes, hypertension, CHF, renal failure, dialysis, disseminated cancer, steroid use for chronic condition, pre-operation systemic infection, emergency case, Na, BUN, Cr, PLT, HCT, INR and operation time. Hazard ratios (HRs) as effect sizes and 95% confidence intervals (CIs) were reported.

Non-linear associations between preoperative WBC counts and thirty-day surgical mortality were assessed by smooth curve fitting using Additive Cox proportional hazard modeling (30). A segmented regression model with two linear segments was employed to investigate the threshold effect in the exposure-outcome relationship based on the smoothing plot. The WBC threshold at which the exposure-outcome relationship changed was determined by recursive testing (31). The optimal exposure-outcome model was identified using a log-likelihood ratio test.

Subgroup analysis using stratified Cox models with stratification based on following factors: sex, age, diabetes, hypertension, COPD, disseminated cancer, steroid usage, preoperative infection, and emergency case. Interaction terms between subgroup indicators and preoperative WBC counts were used to examine subgroup effect modification via likelihood ratio testing. Results reporting adhered to the STROBE guidelines (29, 32).

The statistical analysis was performed using two statistical software packages, namely R (http://www.R-project.org; The R Foundation; version 4.2.0) and EmpowerStats software (www.empowerstats.net, X&Y Solutions, Inc., Boston, MA, United States). A two-sided *p*-value <0.05 was considered significant.

## Results

### Baseline characteristics of participants

The baseline characteristics of the 18,049 cases stratified by preoperative WBC quartiles are depicted in Table 1. The thirty-day surgical mortality was 2.49% (450/18,049). Most parameters significantly differed or trended across quartiles. Patients in higher WBC quartiles showed graded increases in preoperative BUN, HCT, PLT (all *p* < 0.001) and higher rates of smoking, COPD, diabetes, hypertension, disseminated cancer, steroid use and preoperative infection (all *p* < 0.001). In contrast, Na levels declined with rising WBC quartiles (*p* < 0.001). While operation time was shortest for Q4 (*p* < 0.001).

**Table 1:** Baseline characteristics of study population (*N* = 18,049).

WBC counts (quartile)	Q1 (0.10–6.39)	Q2 (6.40–8.48)	Q3 (8.50–11.59)	Q4 (11.60–52.30)	*p*-value
*N* (cases)	4,356	4,651	4,455	4,587	
WBC (10^9/L, Mean ± *SD*)	5.13 ± 0.92	7.36 ± 0.60	9.86 ± 0.89	15.47 ± 3.96	<0.001
BMI (kg/m^2^, Mean ± *SD*)	27.79 ± 5.99	29.01 ± 6.77	29.31 ± 7.04	28.84 ± 6.50	<0.001
Na (mmol/L, Mean ± *SD*)	139.41 ± 2.97	139.09 ± 2.92	138.46 ± 3.15	137.57 ± 3.36	<0.001
BUN (mg/dL, Mean ± *SD*)	15.23 ± 6.01	16.07 ± 6.98	17.75 ± 8.25	20.51 ± 9.55	<0.001
Cr [mg/dL, Median (Q1–Q3)]	0.80 (0.70–0.95)	0.80 (0.70–0.96)	0.80 (0.69–0.95)	0.80 (0.70–0.96)	0.037
HCT (%, Mean ± *SD*)	39.53 ± 4.75	40.45 ± 4.55	40.38 ± 4.79	40.83 ± 5.02	<0.001
PLT (10^9/L, Mean ± *SD*)	214.94 ± 62.69	239.17 ± 67.56	252.55 ± 74.43	268.88 ± 89.06	<0.001
INR (ratio, Mean ± *SD*)	1.02 ± 0.17	1.02 ± 0.17	1.02 ± 0.22	1.03 ± 0.23	0.004
Operation time [minutes, Median (Q1–Q3)]	186.00 (125.00–278.00)	184.00 (123.00–272.50)	182.00 (120.00–267.00)	173.00 (117.00–252.00)	<0.001
Sex, *N* (%)					<0.001
Male	1897 (43.55%)	2,120 (45.58%)	2090 (46.91%)	2,464 (53.72%)	
Female	2,459 (56.45%)	2,531 (54.42%)	2,365 (53.09%)	2,123 (46.28%)	
Race, *N* (%)					<0.001
White	3,082 (70.75%)	3,364 (72.33%)	3,167 (71.09%)	3,193 (69.61%)	
Black	332 (7.62%)	281 (6.04%)	298 (6.69%)	307 (6.69%)	
Asian	145 (3.33%)	153 (3.29%)	103 (2.31%)	123 (2.68%)	
Native	26 (0.60%)	34 (0.73%)	30 (0.67%)	19 (0.41%)	
Unknown	771 (17.70%)	819 (17.61%)	857 (19.24%)	945 (20.60%)	
Age ranges (years), *N* (%)					<0.001
18–40	780 (17.91%)	802 (17.24%)	691 (15.51%)	638 (13.91%)	
41–60	1,859 (42.68%)	1,899 (40.83%)	1,858 (41.71%)	1,883 (41.05%)	
61–80	1,595 (36.62%)	1,804 (38.79%)	1,733 (38.90%)	1,910 (41.64%)	
>80	122 (2.80%)	146 (3.14%)	173 (3.88%)	156 (3.40%)	
Smoking status, *N* (%)					<0.001
No	3,832 (87.97%)	3,835 (82.46%)	3,487 (78.27%)	3,394 (73.99%)	
Yes	524 (12.03%)	816 (17.54%)	968 (21.73%)	1,193 (26.01%)	
Surgical site, *N* (%)					<0.001
Supratentorial	3,353 (76.97%)	3,546 (76.24%)	3,549 (79.66%)	3,717 (81.03%)	
Infratentorial or posterior fossa	918 (21.07%)	1,008 (21.67%)	833 (18.70%)	815 (17.77%)	
Sellar region	66 (1.52%)	73 (1.57%)	56 (1.26%)	43 (0.94%)	
Others	19 (0.44%)	24 (0.52%)	17 (0.38%)	12 (0.26%)	
Functional health status, *N* (%)					0.016
Independent	4,169 (95.71%)	4,459 (95.87%)	4,217 (94.66%)	4,362 (95.09%)	
Partially dependent	151 (3.47%)	148 (3.18%)	176 (3.95%)	186 (4.05%)	
Totally dependent	20 (0.46%)	27 (0.58%)	30 (0.67%)	15 (0.33%)	
Unknown	16 (0.37%)	17 (0.37%)	32 (0.72%)	24 (0.52%)	
Severe COPD, *N* (%)					<0.001
No	4,228 (97.06%)	4,482 (96.37%)	4,222 (94.77%)	4,297 (93.68%)	
Yes	128 (2.94%)	169 (3.63%)	233 (5.23%)	290 (6.32%)	
Diabetes, *N* (%)					<0.001
No	3,959 (90.89%)	4,081 (87.74%)	3,865 (86.76%)	4,019 (87.62%)	
Yes (Insulin)	140 (3.21%)	202 (4.34%)	218 (4.89%)	223 (4.86%)	
Yes (Oral)	257 (5.90%)	368 (7.91%)	372 (8.35%)	345 (7.52%)	
Hypertension, *N* (%)					<0.001
No	2,922 (67.08%)	2,901 (62.37%)	2,682 (60.20%)	2,641 (57.58%)	
Yes	1,434 (32.92%)	1750 (37.63%)	1773 (39.80%)	1946 (42.42%)	
Congestive heart failure, *N* (%)					0.258
No	4,344 (99.72%)	4,641 (99.78%)	4,437 (99.60%)	4,568 (99.59%)	
Yes	12 (0.28%)	10 (0.22%)	18 (0.40%)	19 (0.41%)	
Renal failure, *N* (%)					0.197
No	4,354 (99.95%)	4,649 (99.96%)	4,452 (99.93%)	4,580 (99.85%)	
Yes	2 (0.05%)	2 (0.04%)	3 (0.07%)	7 (0.15%)	
Dialysis, *N* (%)					0.076
No	4,346 (99.77%)	4,636 (99.68%)	4,446 (99.80%)	4,565 (99.52%)	
Yes	10 (0.23%)	15 (0.32%)	9 (0.20%)	22 (0.48%)	
Disseminated cancer, *N* (%)					<0.001
No	3,637 (83.49%)	3,834 (82.43%)	3,419 (76.75%)	3,193 (69.61%)	
Yes	719 (16.51%)	817 (17.57%)	1,036 (23.25%)	1,394 (30.39%)	
Steroid use for chronic condition, *N* (%)					<0.001
No	3,939 (90.43%)	4,077 (87.66%)	3,667 (82.31%)	3,637 (79.29%)	
Yes	417 (9.57%)	574 (12.34%)	788 (17.69%)	950 (20.71%)	
Preoperative systemic infection, *N* (%)					<0.001
No	4,303 (98.78%)	4,600 (98.90%)	4,349 (97.62%)	4,128 (89.99%)	
SIRS	50 (1.15%)	46 (0.99%)	96 (2.15%)	433 (9.44%)	
Sepsis	3 (0.07%)	4 (0.09%)	8 (0.18%)	18 (0.39%)	
Septic Shock	0 (0.00%)	1 (0.02%)	2 (0.04%)	8 (0.17%)	
Emergency case, *N* (%)					<0.001
No	4,183 (96.03%)	4,391 (94.41%)	4,102 (92.08%)	4,184 (91.21%)	
Yes	173 (3.97%)	260 (5.59%)	353 (7.92%)	403 (8.79%)	
Thirty-day mortality events, *N* (%)					<0.001
No	4,297 (98.65%)	4,574 (98.34%)	4,333 (97.26%)	4,395 (95.81%)	
Yes	59 (1.35%)	77 (1.66%)	122 (2.74%)	192 (4.19%)	

WBC, white blood cells; BMI, body-mass index; Na, serum sodium; BUN, blood urea nitrogen; Cr, creatine; HCT, hematocrit; PLT, platelet; INR, International normalized ratio; COPD, chronic obstructive pulmonary disease; SD, standard deviation.

Furthermore, higher WBC categories contained progressively more high-risk subgroups, including larger proportions of males, non-White ethnicities, elderly patients (age > 60 years), supratentorial tumors, and emergent cases (all *p* < 0.001). Functional impairment also differed significantly, being more common in higher WBC quartiles (*p* = 0.016).

### The results of univariate regression analysis

The prognostic indicator for thirty-day surgical mortality assessed by univariate analysis are shown in Supplementary Table S1. Several preoperative factors were significantly associated. Each WBC count unit increase conferred a 7.7% rise in surgical mortality (HR = 1.077, 95%CI: 1.062, 1.093), among the strongest associations seen. Higher BUN, Cr, and INR also increased mortality risk (all *p* < 0.001). By contrast, lower Na, HCT, PLT and operation time portended higher mortality risk (all *p* < 0.005). In terms of demographics, female sex conferred better prognosis than male (*p* < 0.001). Mortality progressed higher with older age (*p* < 0.001). The comorbid conditions including functional impairment, COPD, diabetes, hypertension, CHF, renal issues, metastatic cancer, preoperative infection, steroid usage and emergency cases were also significantly associated with increased mortality risk (all *p* < 0.01). Whereas BMI, race, smoking status, and surgical site had no significant association with mortality risk (all *p* > 0.05).

### The results of multivariate regression analysis

Association between preoperative WBC counts and thirty-day surgical mortality from multivariate analysis is depicted in Table 2. The unadjusted crude model shows a 7.7% rise in thirty-day mortality risk per unit increase in WBC counts (HR = 1.077, 95%CI: 1.062, 1.093). The Model I (minimally adjusted for age and sex) showed an attenuated but significant 6.9% higher risk per unit rise in WBC (HR = 1.069, 95%CI: 1.054, 1.085). The Model II (fully adjusted for 20 confounders) revealed a 5.7% higher thirty-day mortality risk per unit rise in WBC (HR = 1.057, 95%CI: 1.040, 1.076). The distribution of 95%CI indicate a reliable association between preoperative WBC counts and thirty-day surgical mortality.

**Table 2:** The multivariate analysis of the association between preoperative WBC counts and thirty-day surgical mortality.

Exposure	Crude model		Model I		Model II	
	HR (95% CI)	*p*-value	HR (95% CI)	*p*-value	HR (95% CI)	*p*-value
WBC	1.077 (1.062, 1.093)	<0.0001	1.069 (1.054, 1.085)	<0.0001	1.057 (1.040, 1.076)	<0.0001
WBC (quartile)						
Q1 (0.10–6.39)	Ref		Ref		Ref	
Q2 (6.40–8.48)	1.224 (0.872, 1.718)	0.2432	1.181 (0.841, 1.657)	0.3376	1.219 (0.865, 1.719)	0.2573
Q3 (8.50–11.59)	2.036 (1.492, 2.778)	<0.0001	1.899 (1.392, 2.593)	<0.0001	1.704 (1.235, 2.351)	0.0012
Q4 (11.60–52.30)	3.136 (2.343, 4.199)	<0.0001	2.834 (2.115, 3.798)	<0.0001	2.417 (1.760, 3.320)	<0.0001
*P* for trend	<0.001		<0.001		<0.001	

WBC, white blood cells; HR, hazard ratio; 95% CI, 95% confidence interval; Ref, reference. Crude model (non-adjusted model): adjusted for none. Model I (minimally adjusted model): adjusted for sex and age ranges. Model II (fully adjusted model): adjusted for sex, age ranges, functional status, COPD, diabetes, hypertension, CHF, renal failure, dialysis, disseminated cancer, steroid use, preoperative infection, emergency case, Na, BUN, Cr, HCT, PLT, INR, and operation time.

### Non-linear relationship between WBC counts and thirty-day mortality

**Figure 2:**
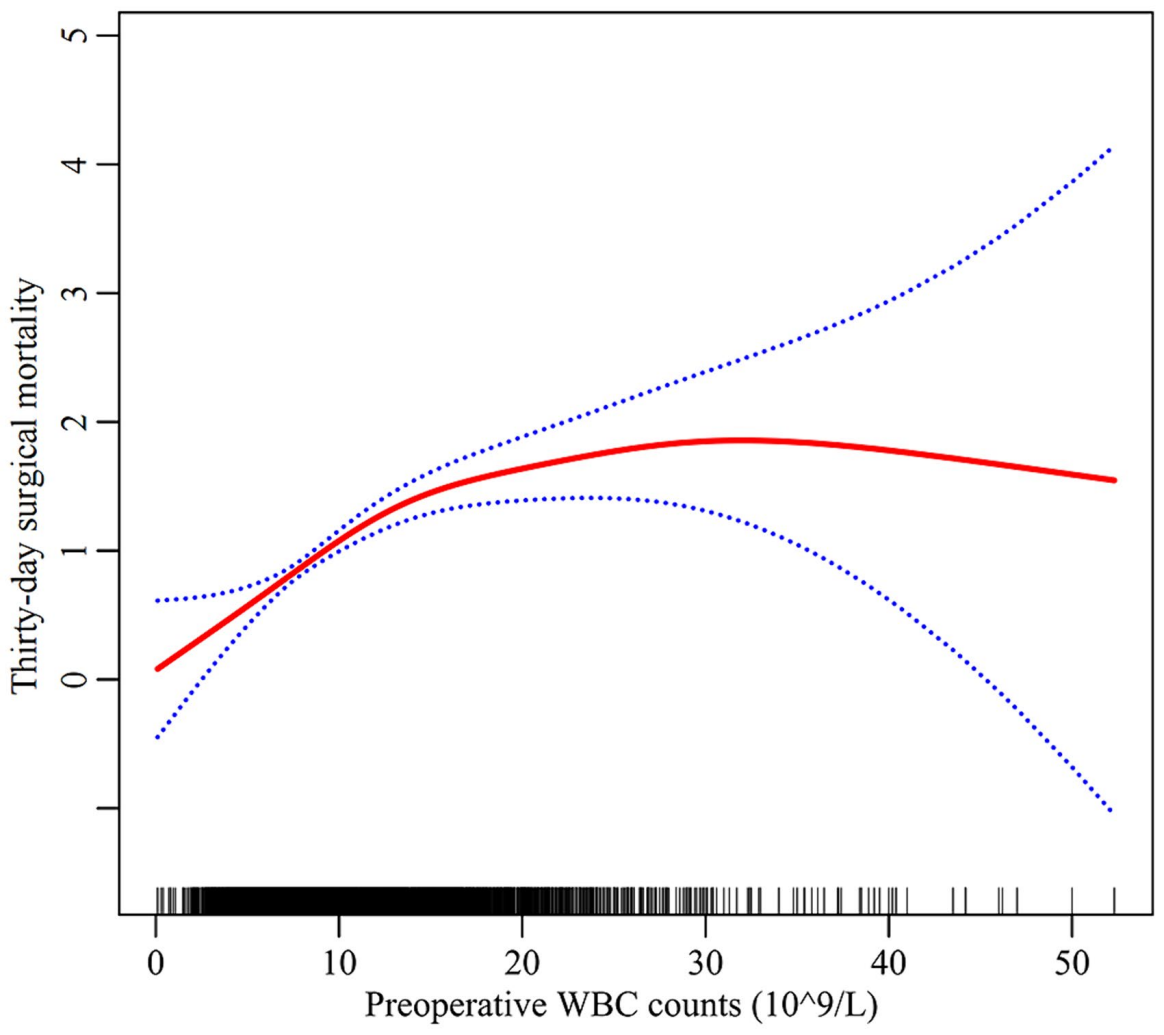
The non-linear relationship between preoperative WBC counts and thirty-day surgical mortality. Red line: thirty-day surgical mortality; Blue line: 95%CI Note: the model adjusted for all covariates in line with the multivariate analysis.

As depicted in Figure 2, preoperative WBC counts and thirty-day surgical mortality had a non-linear relationship. A segmented regression model with two linear segments identified 13.6 × 10^9/L as the inflection point of WBC counts (depicted in Table 3). Below this threshold, each WBC unit increase conferred a 11.7% rise in surgical mortality (HR = 1.117, 95%CI: 1.077, 1.158). However, above the cutoff, the thirty-day surgical mortality risk plateaued without significant further escalation (HR = 1.015, 95%CI: 0.982, 1.050). Log likelihood ratio testing confirmed superior fit with the non-linear two-segmented model versus standard linear model (*p* < 0.001).

**Table 3:** The results of the two-segmented linear regression analysis.

Outcome	Thirty-day surgical mortality	
	HR (95% CI)	*P*-value
Standard linear model	1.057 (1.040, 1.076)	<0.0001
Two-segmented linear model		
Inflection point of WBC counts	13.6	
<13.6	1.117 (1.077, 1.158)	<0.0001
>13.6	1.015 (0.982, 1.050)	0.3690
*P*-value for the log likelihood ratio test	<0.001	

HR, hazard ratio; 95% CI, 95% confidence interval. The model adjusted for sex, age ranges, functional status, COPD, diabetes, hypertension, CHF, renal failure, dialysis, disseminated cancer, steroid use, preoperative infection, emergency case, Na, BUN, Cr, HCT, PLT, INR and Operation time.

### The results of subgroup analysis and interaction testing

Subgroup analysis was performed to the detect the influence of other stratified factors on the trend of exposure-outcome association (depicted in Table 4). The prognostic impact of preoperative WBC counts was generally consistent across these clinical subgroups except steroid use for chronic condition. A significant interaction was seen with steroid use for chronic condition (*P* for interaction = 0.002). In non-steroid users, each WBC unit rise conferred a 7.2% higher thirty-day surgical mortality (HR = 1.072, 95%CI: 1.051, 1.093). Whereas no prognostic value of WBC counts was evident in steroid users (HR = 1.029, 95%CI: 0.995, 1.063). No significant effect modifications on the exposure-outcome association were observed in different groups of sex, age, diabetes, hypertension, COPD, disseminated cancer, preoperative infection and emergency case (all *P* for interaction >0.05).

**Table 4:** The results of subgroup analysis and interaction testing.

Characteristic	N	HR (95% CI)	*p*-value	*P* for interaction
Sex				0.556
Male	8,571	1.050 (1.026, 1.074)	<0.0001	
Female	9,478	1.067 (1.039, 1.095)	<0.0001	
Age ranges				0.390
18–60	10,410	1.065 (1.035, 1.096)	<0.0001	
61–80	7,042	1.053 (1.027, 1.079)	<0.0001	
>80	597	1.036 (0.982, 1.093)	0.1963	
Diabetes				0.166
No	15,924	1.058 (1.038, 1.078)	<0.0001	
Yes (Insulin and Oral)	2,125	1.068 (1.022, 1.116)	0.0037	
Severe COPD				0.419
No	17,229	1.061 (1.042, 1.080)	<0.0001	
Yes	820	0.988 (0.919, 1.061)	0.7313	
Hypertension				0.248
No	11,146	1.055 (1.027, 1.083)	<0.0001	
Yes	6,903	1.060 (1.037, 1.085)	<0.0001	
Disseminated cancer				0.113
No	14,083	1.057 (1.034, 1.081)	<0.0001	
Yes	3,966	1.051 (1.023, 1.079)	0.0003	
Steroid use for chronic condition				0.002
No	15,320	1.072 (1.051, 1.093)	<0.0001	
Yes	2,729	1.029 (0.995, 1.063)	0.0944	
Preoperative systemic infection				0.091
No	17,380	1.059 (1.040, 1.078)	<0.0001	
SIRS/Sepsis/Septic Shock	669	1.046 (0.997, 1.097)	0.0645	
Emergency case				0.293
No	16,860	1.054 (1.034, 1.075)	<0.0001	
Yes	1,189	1.083 (1.037, 1.130)	0.0003	

HR, hazard ratio; 95% CI, 95% confidence interval. The model adjusted for sex, age range, functional status, COPD, diabetes, hypertension, CHF, renal failure, dialysis, disseminated cancer, steroid use, preoperative infection, emergency case, Na, BUN, Cr, HCT, PLT, INR and Operation time. The model adjusted for all above variables except the corresponding stratification variable.

### Steroid usage difference in the non-linear association

**Figure 3:**
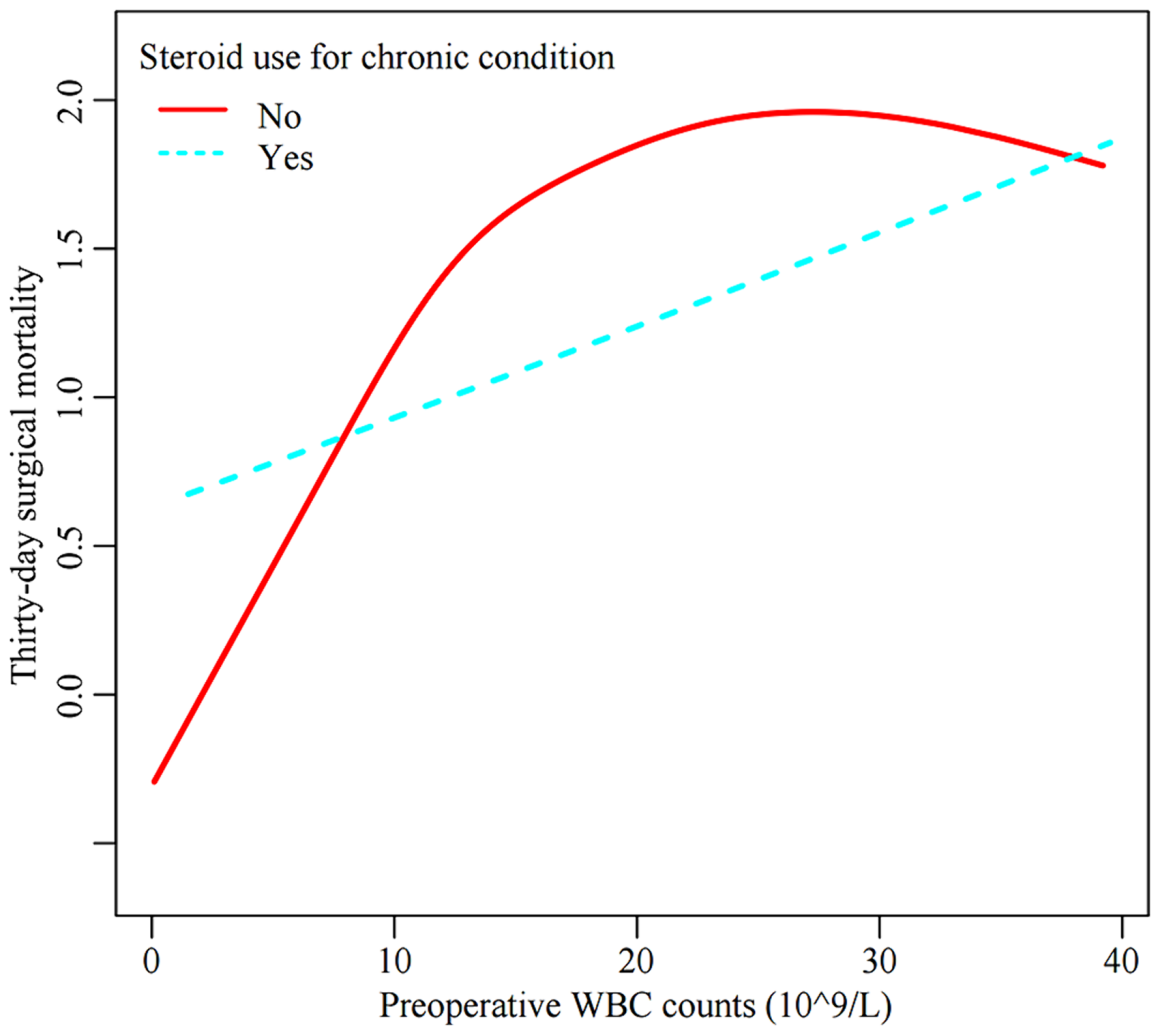
The effect modification of steroid usage status on the non-linear association between preoperative WBC counts and thirty-day surgical mortality. Note: The model adjusted for all covariates in line with the multivariate analysis except steroid usage.

We then explored the effect modifications of steroid usage status on the non-linear association between preoperative WBC counts and thirty-day surgical mortality. Steroid usage status was used as a stratification factor to perform smooth curve fitting and segmented linear regression analysis. As depicted in Figure 3, a non-linear exposure-outcome association was present for non-steroid users but not steroid users. As depicted in Table 5, the inflection point of WBC counts was 13.6 × 10^9/L. Below this threshold, each WBC unit increase conferred a 15.8% rise in thirty-day mortality for non-steroid users (HR = 1.158, 95%CI: 1.108, 1.210). Above this threshold, the risk plateaued without significant further escalation (HR = 1.009, 95%CI: 0.966, 1.055). No significant linear or non-linear exposure-outcome association was observed for steroid users. These results suggest that the non-linear WBC-mortality association differed between steroid and non-steroid users.

**Table 5:** The results of the two-segmented linear regression analysis stratified by steroid usage status.

Outcome	Thirty-day surgical mortality (HR, 95% CI, *P*-value)	
Steroid use	No (*N* = 15,320)	Yes (*N* = 2,729)
Standard linear model	1.072 (1.051, 1.093) <0.0001	1.029 (0.995, 1.063) 0.0944
Two-segmented linear model		
Inflection point of WBC counts	13.6	13.6
<13.6	1.158 (1.108, 1.210) <0.0001	1.021 (0.954, 1.093) 0.5423
>13.6	1.009 (0.966, 1.055) 0.6796	1.034 (0.982, 1.088) 0.2078
*P*-value for the log likelihood ratio test	<0.001	0.815

HR, hazard ratio; 95% CI, 95% confidence interval. The model adjusted for sex, age range, functional status, COPD, diabetes, hypertension, CHF, renal failure, dialysis, disseminated cancer, preoperative infection, emergency case, Na, BUN, Cr, HCT, PLT, INR and Operation time.

### Sensitivity analysis

Our sensitivity analysis evaluated result robustness. As depicted in Table 2, preoperative WBC counts were categorized into quartile. In categorical analysis with the categorical-transformed WBC counts, the upper two quartiles showed higher HRs (Q3 HR = 1.704, 95%CI: 1.235, 2.351; Q4 HR = 2.417, 95%CI: 1.760, 3.320) compared to the lowest quartile (Q1). Significant trends persisted across quartiles (*p* < 0.001). Furthermore, we reanalyzed the data after excluding 3,009 cases with missing data from 18,049 patients. The thirty-day mortality in the complete cases was 2.71% (407/15,040). In Supplementary Table S2, the multivariate analysis revealed a 5.4% higher thirty-day mortality risk per unit rise in WBC counts (HR = 1.054, 95%CI: 1.035, 1.073). In Supplementary Table S3, the segmented linear regression analysis identified a non-linear exposure-outcome association, showing a 10.7% higher thirty-day mortality risk per unit rise in WBC counts below the threshold (HR = 1.107, 95%CI: 1.064, 1.150) and a plateaued risk above the threshold (HR = 1.017, 95%CI: 0.982, 1.052). These results were consistent with our prior analysis, confirming the robustness of our findings.

## Discussion

Analyzing ACS NSQIP data (2012–2015), this large retrospective cohort study of 18,049 cases explored associations between preoperative WBC counts and thirty-day surgical mortality after craniotomy in adult intracranial tumor patients. Our findings indicate that elevated preoperative WBC counts has an independent and non-linear association with increased thirty-day surgical mortality risk, with a significant threshold effect at a WBC count of 13.6 × 10^9/L. Below this threshold, higher WBC counts was associated with elevated thirty-day mortality risk, while the mortality risk plateaued above this level. Furthermore, different steroid usage status has significant effect modification on this association (*P* for interaction = 0.002). This exposure-outcome association was only present for non-steroid users but not steroid users.

Prior studies have demonstrated a connection between WBC counts and prognosis in diverse diseases, establishing it as a biomarker for mortality risk in conditions such as acute cerebral infarction (20), aortic dissection (33), acute myocardial infarction (34), bladder cancer surgery (35), sepsis (36), and lung cancer (37). Collectively, these findings highlight the WBC count’s role as a predictive indicator for patient outcomes in various clinical contexts. Earlier research has identified preoperative WBC count as an independent prognostic indicator of long-term postoperative outcomes in glioblastoma patients (38). However, the relationship between preoperative WBC counts and short-term outcomes after intracranial tumor resection had not been explored, which our study aimed to investigate. Consistent with previous findings, our study indicates that preoperative WBC counts can independently predicts short-term prognosis in adult intracranial tumor patients without steroid usage undergoing craniotomy, significantly associating elevated WBC levels with an heightened thirty-day surgical mortality risk.

However, the exact mechanisms through which WBC count affects patient outcomes are not yet fully understood. Current research leads us to hypothesize multiple contributing factors: An increased preoperative WBC count could indicate systemic inflammation, as preoperative SIRS has been established as a major risk predictor for postoperative sepsis and septic shock – conditions linked to higher thirty-day surgical mortality (10). The involved pathophysiological mechanisms likely involve WBC-driven inflammatory pathways that exacerbate tissue damage and disease progression (26). Additionally, WBC counts are established as independent predictors of cardiovascular, cerebrovascular, and thrombotic event risks (39–41). Therefore, higher preoperative WBC counts may signal an elevated risk for these events, contributing to increased mortality. Furthermore, research has shown a association between WBC counts and cancer staging (42, 43). A higher preoperative WBC count may imply a more advanced tumor grade and stage, correlated with a worse prognosis. Meanwhile, given that steroids have the ability to cause WBC to demarginate from blood vessel walls into circulation and increase peripheral blood WBC counts, the WBC counts in adult intracranial tumor patients using steroids usually cannot accurately reflect their inflammatory status *in vivo*. These factors may collectively account for the underlying mechanisms linking WBC counts with short-term postoperative outcomes. Meanwhile, regarding the phenomenon that the association between WBC counts and thirty-day mortality plateaued above 13.6 × 10^9/L, we speculate the following reasons: when the WBC counts exceed a certain threshold, the inflammatory response may have reached its maximum extent, and further elevations in WBC counts may not confer additional harm. Moreover, extremely high WBC counts may indicate advanced-stage tumors with already poor prognosis, thus obscuring any further impact of WBC count on mortality risk. However, given the retrospective nature of this study, we cannot draw definitive conclusions, and further studies are needed to elucidate the underlying mechanisms.

Overall, our findings align with prior evidence across settings showing prognostic value for preoperative WBC counts. Notably, this analysis provides first validation in an American population that preoperative WBC counts independently predicts short-term outcomes of intracranial tumor patients after craniotomy, especially in non-steroid users. This offers a readily available biomarker that aids in the identification of high-risk patients, thereby enhancing preoperative evaluation and preparation to facilitate personalized treatment. Furthermore, our findings lay a foundation for future exploration into the potential application of preoperative WBC counts in prognostic assessments following intracranial tumor surgery.

Our study has the following strengths: Firstly, the study included 18,049 patients, providing a large sample size and solid dataset for analysis. Secondly, there was little missing information on covariates, allowing adjustment for numerous confounders and multi-model effects assessment. Thirdly, sensitivity analysis were conducted to confirm the robustness of results. Fourthly, Smooth curve fitting in conjunction with segmented linear regression modeling and threshold analysis were employed for exploring the non-linear exposure-outcome relationship. Fifthly, subgroup analysis and interaction testing were performed to assess predictive effects across populations. Overall, this study employed a rigorous approach according to the STROBE guidelines, consistent with prior studies and showcasing dependable findings.

Our study has the following limitations: Firstly, as a retrospective cohort study, it can only establish association. Prior studies have revealed that certain other factors, such as platelet counts (11), hematocrit (12), and serum sodium (13), were also associated with postoperative short-term outcomes, suggesting that WBC counts may represent just one of many surrogate parameters for poor prognosis. Given our purely associative results, we cannot establish causality between WBC counts and thirty-day mortality. Secondly, as a secondary analysis based on publicly database, it could not exclude the effect of some unmeasured confounding factors such as genetics, environment and tumor stage. Thirdly, the original database lacked data on WBC subtypes, so the neutrophil to lymphocyte ratio cannot be calculated and used to analyze its impact on outcome indicators. Fourthly, this study was limited to the U.S. population, thereby limiting it’s generalizability to other populations due to differences in baseline characteristics and treatment patterns. Fifthly, the primary outcome measure was thirty-day surgical mortality, the association between WBC counts and long-term survival could not be evaluated. Sixthly, some statistically significant differences in our findings may be caused by the large sample size and multiple testing. For example, many of the significant differences across WBC count quartiles in some factors such as Cr, INR, operation time are actually subtle and not clinically relevant. Seventh, our findings are based on a single registry database, thus should be considered as exploratory. They have not been validated properly in other independent cohorts. As such, further researches in future are warranted to improve the reliability and generalizability of our results, and establish causal mechanisms through robust experimental designs.

## Conclusion

For the first time in a large U.S. cohort, this study identified an independent and non-linear association between preoperative WBC counts and thirty-day surgical mortality in adult non-steroid use patients undergoing craniotomy for intracranial tumors. Elevated WBC counts were significantly associated with increased thirty-day mortality risk, while mortality risk stabilized without significant further escalation above a WBC threshold of 13.6 × 10^9/L. This easily accessible predictor can identify intracranial tumor patients facing high postoperative mortality hazards via elevated preoperative WBC levels. Such data may optimize surgical risk profiling and guide WBC management to mitigate thirty-day mortality risk. However, given the retrospective cohort design, causal links and generalizability remain to be established in further studies.

## Data availability statement

Publicly available datasets were analyzed in this study. This data can be found at: https://doi.org/10.1371/journal.pone.0235273.s001.

## Ethics statement

The requirement of ethical approval was waived by Ethics Committee of The First People’s Hospital of Xiaoshan District for the studies involving humans because this study represents a secondary analysis utilizing a published public database with retrospective analytical nature. The studies were conducted in accordance with the local legislation and institutional requirements. The Ethics Committee/Institutional Review Board also waived the requirement of written informed consent for participation from the participants or the participants’ legal guardians/next of kin because this study is based on a de-identified database, the original personal information was anonymous.

## Author contributions

ZG: Data curation, Writing – original draft, Formal analysis, Investigation, Methodology. CH: Data curation, Formal analysis, Writing – original draft, Investigation. SF: Data curation, Writing – original draft, Formal analysis. JG: Writing – original draft, Software. WD: Project administration, Supervision, Writing – review & editing, Resources.
