# Supplementary material for: Association between preoperative white blood cell counts and thirty-day surgical mortality after craniotomy in adult intracranial tumor patients
Source: Front Neurol. 2024 Jul 5;15:1394568. doi: 10.3389/fneur.2024.1394568 (PMC11259970; doi:10.3389/fneur.2024.1394568)
Supplement: Supplementary file 1 [file Table_1.docx]

Supplementary Material

# Supplementary Data

**Supplemental Table 1** The univariate analysis of thirty-day surgical mortality

|  | Statistics | HR (95%CI) | P-value |
| --- | --- | --- | --- |
| WBC (Mean ± SD) | 9.501 ± 4.402 | 1.077 (1.062, 1.093) | <0.0001 |
| BMI (Mean ± SD) | 28.746 ± 6.614 | 0.987 (0.973, 1.002) | 0.0929 |
| Na (Mean ± SD) | 138.624 ± 3.183 | 0.908 (0.885, 0.932) | <0.0001 |
| BUN (Mean ± SD) | 17.410 ± 8.082 | 1.032 (1.028, 1.037) | <0.0001 |
| Cre (Mean ± SD) | 0.862 ± 0.447 | 1.223 (1.113, 1.344) | <0.0001 |
| HCT (Mean ± SD) | 40.311 ± 4.804 | 0.922 (0.907, 0.938) | <0.0001 |
| PLT (Mean ± SD) | 244.174 ± 76.760 | 0.998 (0.997, 0.999) | 0.0017 |
| INR (Mean ± SD) | 1.024 ± 0.200 | 1.353 (1.166, 1.571) | <0.0001 |
| Operation time (Mean ± SD) | 211.330 ± 131.669 | 0.998 (0.998, 0.999) | 0.0001 |
| Sex, N (%) |  |  |  |
| male | 8571 (47.487%) | Ref |  |
| female | 9478 (52.513%) | 0.646 (0.536, 0.779) | <0.0001 |
| Race, N (%) |  |  |  |
| White | 12806 (70.951%) | Ref |  |
| Black | 1218 (6.748%) | 0.900 (0.607, 1.333) | 0.5986 |
| Asian | 524 (2.903%) | 0.693 (0.357, 1.344) | 0.2777 |
| Native | 109 (0.604%) | 1.121 (0.360, 3.495) | 0.8439 |
| Unknown | 3392 (18.793%) | 1.138 (0.905, 1.431) | 0.2690 |
| Age ranges, N (%) |  |  |  |
| 18-40 | 2911 (16.128%) | Ref |  |
| 41-60 | 7499 (41.548%) | 2.750 (1.722, 4.393) | <0.0001 |
| 61-80 | 7042 (39.016%) | 4.876 (3.088, 7.698) | <0.0001 |
| >80 | 597 (3.308%) | 14.238 (8.545, 23.724) | <0.0001 |
| Smoking status, N (%) |  |  |  |
| No | 14548 (80.603%) | Ref |  |
| Yes | 3501 (19.397%) | 1.128 (0.901, 1.414) | 0.2940 |
| Surgical site, N (%) |  |  |  |
| Supratentorial | 14165 (78.48%) | Ref |  |
| Infratentorial or posterior fossa | 3574 (19.80%) | 0.96 (0.76, 1.21) | 0.7303 |
| Sellar region | 238 (1.32%) | 0.49 (0.16, 1.54) | 0.2244 |
| Others | 72 (0.40%) | 0.55 (0.08, 3.88) | 0.5447 |
| Functional health status, N (%) |  |  |  |
| Independent | 17207 (95.335%) | Ref |  |
| Partially Dependent | 661 (3.662%) | 3.927 (2.959, 5.212) | <0.0001 |
| Totally Dependent | 92 (0.510%) | 7.020 (4.038, 12.204) | <0.0001 |
| Unknown | 89 (0.493%) | 4.299 (2.134, 8.660) | <0.0001 |
| Severe COPD, N (%) |  |  |  |
| No | 17229 (95.457%) | Ref |  |
| Yes | 820 (4.543%) | 2.489 (1.840, 3.367) | <0.0001 |
| Diabetes, N (%) |  |  |  |
| No | 15924 (88.226%) | Ref |  |
| Yes (Insulin) | 783 (4.338%) | 2.530 (1.850, 3.460) | <0.0001 |
| Yes (Oral) | 1342 (7.435%) | 1.527 (1.124, 2.076) | 0.0068 |
| Hypertension, N (%) |  |  |  |
| No | 11146 (61.754%) | Ref |  |
| Yes | 6903 (38.246%) | 2.208 (1.831, 2.662) | <0.0001 |
| Congestive heart failure, N (%) |  |  |  |
| No | 17990 (99.673%) | Ref |  |
| Yes | 59 (0.327%) | 6.780 (3.504, 13.117) | <0.0001 |
| Renal failure, N (%) |  |  |  |
| No | 18035 (99.922%) | Ref |  |
| Yes | 14 (0.078%) | 9.171 (2.946, 28.544) | 0.0001 |
| Dialysis, N (%) |  |  |  |
| No | 17993 (99.690%) | Ref |  |
| Yes | 56 (0.310%) | 6.243 (3.103, 12.563) | <0.0001 |
| Disseminated cancer, N (%) |  |  |  |
| No | 14083 (78.026%) | Ref |  |
| Yes | 3966 (21.974%) | 2.829 (2.348, 3.408) | <0.0001 |
| Steroid use for chronic condition, N (%) |  |  |  |
| No | 15320 (84.880%) | Ref |  |
| Yes | 2729 (15.120%) | 2.307 (1.882, 2.829) | <0.0001 |
| Preoperative systemic infection, N (%) |  |  |  |
| No | 17380 (96.293%) | Ref |  |
| SIRS | 625 (3.463%) | 2.066 (1.426, 2.993) | 0.0001 |
| Sepsis | 33 (0.183%) | 11.684 (5.804, 23.522) | <0.0001 |
| Septic Shock | 11 (0.061%) | 8.515 (2.122, 34.163) | 0.0025 |
| Emergency case, N (%) |  |  |  |
| No | 16860 (93.412%) | Ref |  |
| Yes | 1189 (6.588%) | 2.761 (2.146, 3.552) | <0.0001 |

WBC: White blood cells; BMI: Body-mass index; Na: Serum sodium; BUN: blood urea nitrogen; Cr: creatine; HCT: hematocrit; PLT: platelet; INR: International normalized ratio; HR, hazard ratio; 95%CI, 95% confidence interval; Ref, reference; SD: standard deviation.

**Supplemental Table 2** The Comparison of multivariate analysis results from two datasets

| Datasets | Mean/Median imputation |  | Complete cases |
| --- | --- | --- | --- |
|  | (N = 18,049) |  | (N = 15,040) |
| Exposure | HR ( 95% CI) P-value |  | HR ( 95% CI ) P-value |
| WBC | 1.057 (1.040, 1.076) <0.00001 |  | 1.054 (1.035, 1.073) <0.00001 |
| WBC(quartile) |  |  |  |
| Q1 (0.10-6.39) | Ref |  | Ref |
| Q2 (6.40-8.48) | 1.219 (0.865, 1.719) 0.25725 |  | 1.185 (0.826, 1.701) 0.35722 |
| Q3 (8.50-11.59) | 1.704 (1.235, 2.351) 0.00116 |  | 1.591 (1.133, 2.234) 0.00730 |
| Q4 (11.60-52.30) | 2.417 (1.760, 3.320) <0.00001 |  | 2.219 (1.585, 3.107) <0.00001 |
| P for trend | <0.001 |  | <0.001 |

WBC: White blood cells; HR, hazard ratio; 95% CI, 95% confidence interval; Ref, reference.

The model fully adjusted for sex, age ranges, functional status, COPD, diabetes, hypertension, CHF, renal failure, dialysis, disseminated cancer, steroid use, preoperative infection, emergency case, Na, BUN, Cr, HCT, PLT, INR and Operation time.

**Supplemental Table 3** The results of two-segmented linear regression analysis after excluding missing data (N=15,040)

| Outcome: | Thirty-day surgical mortality | |
| --- | --- | --- |
|  | HR ( 95% CI ) | P-value |
| Standard linear model | 1.054 (1.035, 1.073) | <0.0001 |
| Two-segmented linear model |  |  |
| Inflection point of WBC counts | 13.6 |  |
| < 13.6 | 1.107 (1.064, 1.150) | <0.0001 |
| > 13.6 | 1.017 (0.982, 1.052) | 0.3520 |
| P-value for the log likelihood ratio test | 0.004 |  |

HR, hazard ratio; 95% CI, 95% confidence interval
The model fully adjusted for sex, age ranges, functional status, COPD, diabetes, hypertension, CHF, renal failure, dialysis, disseminated cancer, steroid use, preoperative infection, emergency case, Na, BUN, Cr, HCT, PLT, INR and Operation time.

**Supplemental Table 4** Surgical site and corresponding CPT codes

| Surgical site | CPT codes Surgical procedures | |
| --- | --- | --- |
| Supratentorial | 61510 | Supratentorial craniotomy for tumor |
|  | 61512 | Supratentorial craniotomy for meningioma |
| Infratentorial or posterior fossa | 61518 | Infratentorial craniotomy for tumor: others |
|  | 61519 | Infratentorial craniotomy for meningioma |
|  | 61520 | Infratentorial craniotomy for tumor: cerebellopontine angle |
|  | 61521 | Infratentorial craniotomy for tumor: midline |
|  | 61526 | Trans labyrinthine approach for cerebellopontine angle tumor |
|  | 61575 | Transoral approach to skull base, brain stem, or upper spinal cord for biopsy, decompression,or excision of lesion |
| Sellar region | 61545 | Craniotomy for craniopharyngioma |
|  | 61546 | Craniotomy for pituitary macroadenoma |
| Others | See details in “[https://doi.org/10.1371/journal.pone.0235273](https://doi.org/10.1371/journal.pone.0235273.s001)” | |
